# Growth and interface formation in CeO_*x*_–Ni(111) inverse model catalysts

**DOI:** 10.1039/d6na00679e

**Published:** 2026-08-13

**Authors:** Raquel Sánchez-Barquilla, Dominic Guttmann, Carlos Morales, Jan Ingo Flege

**Affiliations:** a Applied Physics and Semiconductor Spectroscopy, Brandenburg University of Technology Cottbus-Senftenberg 03046 Cottbus Germany flege@b-tu.de

## Abstract

We investigated the inverse catalyst configuration CeO_*x*_/Ni(111) by combining diffraction, spectroscopic, and imaging techniques to obtain a complete picture of the growth mode. Our results from quantitative X-ray photoelectron spectroscopy, low-energy electron diffraction, and scanning tunneling microscopy reveal a complex system in which the initial oxidation of the Ni substrate plays a fundamental role in the preferred orientation of ceria and its initial reduction. We demonstrate the formation of a NiO(111) interfacial layer between the CeO_*x*_(111) wetting layer and the Ni substrate, while thicker cerium oxide islands nucleate at step edges. Furthermore, we found evidence of oxygen migration and electronic charge transfer at the interface, resulting in a CeO_*x*_/NiO interface rich in Ce^3+^ states. These results highlight the complex structural and electronic interactions established between the film and the substrate, offering deeper insights into the underlying interfacial physics and chemistry of ceria-nickel catalysts.

## Abstract 2

This study reveals the dynamic role of a NiO interface layer in mediating the growth and electronic properties of CeO_*x*_/Ni(111).

## Graphical abstract

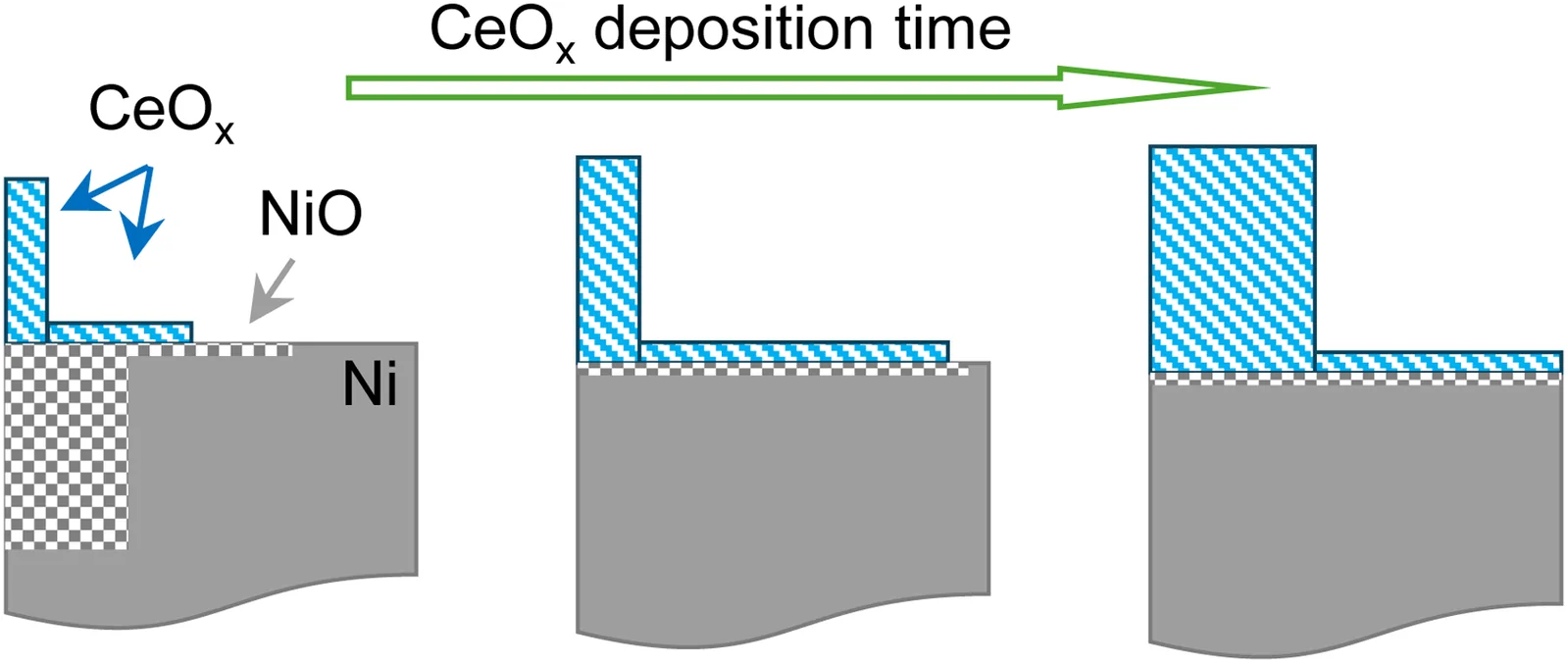

## 1 Introduction

Climate change and fossil fuel depletion are among the most pressing challenges of our time. Although reducing emissions remains crucial, capturing and reusing CO_2_ is equally important.^1^ Primarily released from fossil fuel combustion, CO_2_ is a major contributor to global warming, but when captured from industrial emissions or the atmosphere, it becomes a valuable feedstock. Converting CO_2_ into synthetic fuels enables efficient energy storage, provides an alternative to batteries to stabilize the renewable energy supply, and extends the lifespan of existing power plants and engines, thereby creating a closed carbon cycle.^2^ Additionally, CO_2_-derived hydrocarbons serve as essential building blocks for the chemical industry. Over the last few decades, great efforts have been invested in developing catalysts that improve CO_2_ conversion efficiency and stability, particularly at low temperatures.^2,3^

In this context, nickel particles supported on CeO_2_ are a widely used metal/oxide catalyst, showing high efficiency for CO_2_,^4,5^ methane,^6,7^ and water activation^8^ at relatively low temperatures. This efficiency is attributed to strong metal–support interactions (SMSI)^9,10^ and electronic metal–support interaction (EMSI),^11^ mediated by the electronic influence of ceria, which changes the oxidation state of Ni^0^ particles to Ni^2+^ by generating two Ce^3+^.^4,8,12^ Furthermore, the CO methanation reaction on nickel has been suggested to be phase-dependent, with the active sites being undercoordinated Ni atoms (*e.g.*, steps).^13^ Therefore, controlling this SMSI as well as the atomic arrangement is essential for improving catalytic activity.

Model catalysts based on single crystals provide an indispensable platform to unravel the structural and electronic requirements for optimizing the catalyst's performance in the different reactions. The so-called inverse catalyst architecture,^14–16^ where the metal oxide is deposited on a metallic substrate, provides novel possibilities of studying the role of the oxide and its contribution to the catalytic process. Consequently, it has been discussed as a means to enhance conversion rates through the increased complexity of the SMSI, allowing reactants to interact with the defect sites of the metal oxide nanoparticles, the metallic support, and the metal-oxide interface.^16^ For the CeO_*x*_/Cu case, the inverse system has shown higher catalytic performance than its traditional configuration due to an active oxide/metal interface that presents oxide centers with dynamic chemical properties, forming CeO_2_/Cu_2_O/Cu(111).^10^ In these inverse systems, the growth mechanism of ceria nanoislands and thin films can be significantly influenced by the substrate's characteristics, thereby affecting the resulting morphology, facet orientation, and degree of oxidation. For example, ceria grows mainly in the fluorite (111) orientation on Ru(0001),^17–19^ Au(111),^20,21^ and Pt(111),^22–24^ where the nanoislands exhibit very different sizes and shapes under the same growth conditions that have been associated with the different oxygen potential at the surface of the substrate. On Cu(111), however, the different oxygen partial pressure can tune the cubic CeO_2_ islands from (111) to (100) orientation,^25^ which have been shown to present phase-dependent reducibility under Sm doping.^26^

From the composition point of view, the temperature of the substrate strongly influences the stoichiometry of ceria when deposited on Ni(111),^18^ which again is also highly dependent on the metallic support. For instance, when grown at 700 K, the ceria film is fully oxidized on Ru(0001) but slightly reduced on Ni(111).^18^ Thus, this brief overview demonstrates that to establish a relationship between catalytic properties and SMSI, it is crucial to comprehend the interaction between CeO_*x*_ deposits and metallic substrates, which ultimately determines the structural growth of CeO_2_ layers as well as the initial oxidation states of the film and substrate.

Despite the widespread use of traditional Ni/ceria catalysts, the inverse ceria/Ni architecture remains largely unexplored. This is likely due to the lower structural order and compositional stability of CeO_2_ films grown on Ni(111) compared to other substrates, such as Ru(0001).^18^ Thus, investigating this system requires spectroscopic techniques, such as X-ray photoelectron spectroscopy (XPS), which are less dependent on crystalline order. The key point, however, is to correlate these findings, under certain growth conditions, with insights from scanning probe microscopy and surface diffraction techniques, the latter enabling direct access to structural information on the atomic scale. Here, we present the first comprehensive study of the ceria–nickel(111) inverse model catalyst by targeting the growth of ceria islands on Ni(111) at moderate temperatures, along with the structural characterization using three complementary techniques: low-energy electron diffraction (LEED), scanning tunneling microscopy (STM), and inelastic peak shape analysis (IPSA) *via* XPS. This structural information is directly correlated with the chemical oxidation states of Ce and Ni, as well as the established electronic interaction between the film and the substrate.

## 2 Experimental details

**Fig. 1:**
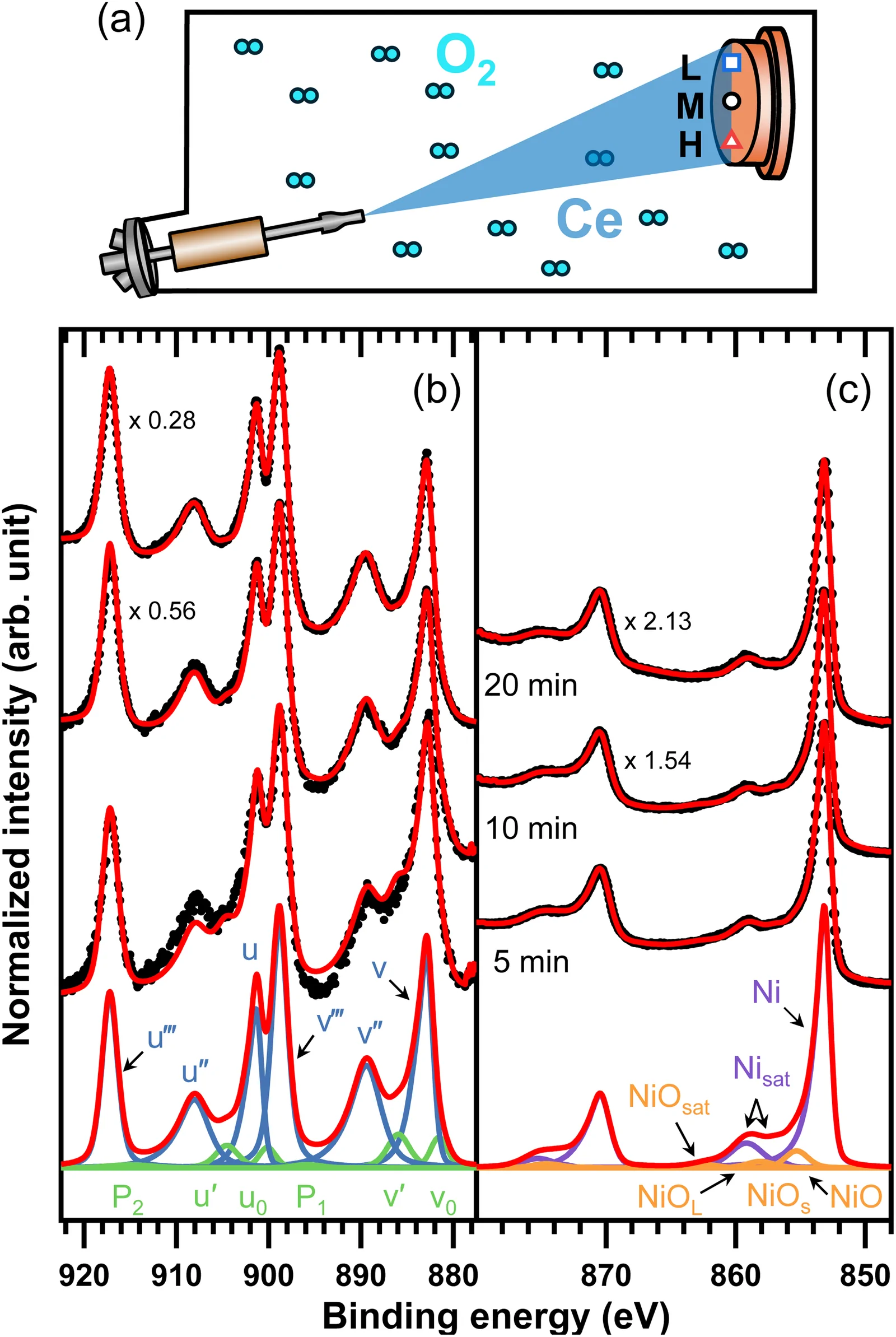
(a) Schematic representation of the evaporation geometry. H, M, and L positions are indicated on the sketched crystal. Light blue circles indicate the oxygen atmosphere, and the Ce evaporation is indicated by the colored dark blue area. XPS spectra at the (b) Ce 3d and (c) Ni 2p regions for the three deposition steps at the medium (M) position. The fit models for the deposition after 20 min of both regions are provided, showing the Ce^3+^ (green), Ce^4+^ (blue), metallic nickel (purple), and NiO (yellow) components.

A 10 mm diameter Ni(111) single crystal from Surface Preparation Laboratory (SPL), with a nominal orientation better than 0.1°, was used as a substrate. The crystal was cleaned *in situ* by successive cycles of Ar^+^ sputtering (*P*_Ar^+^_ = 1 × 10^−5^ mbar) and annealing up to 800 °C. The substrate was then flashed to 1100 °C several times to obtain flat surfaces.^27,28^ Crystal cleanliness and crystalline order were confirmed by XPS and LEED. The growths were carried out using a commercial e-beam evaporator (Focus EFM 3) with a molybdenum crucible loaded with metallic cerium (Alfa Aesar, 99.9%). The three evaporations were performed in an oxygen atmosphere of 1 × 10^−7^ mbar and 500 °C in three steps of 5, 5, and 10 minutes, without cleaning the sample in between. After each growth, the sample was cooled down in oxygen to avoid ceria reduction and was characterized by XPS and LEED in ultra-high vacuum (UHV) conditions. The evaporator was calibrated to an average deposition rate of 1 ML min^−1^. The cerium deposition was performed with the evaporator facing one edge of the sample to obtain a ceria thickness gradient for each deposition step. Then, three different areas corresponding to different ceria thicknesses, with an acceptance area of 2 mm in diameter, were measured by XPS. These positions are referred to as low (L), medium (M), and high (H) cerium deposition, and their position on the sample surface relative to the evaporator is schematically indicated in Fig. 1. The LEED measurements are taken at the center of the single crystal, M position.

LEED and STM measurements were performed using a commercial ErLEED and Aarhus 150 from SPECS. Images were processed using the WXSM software.^29^ XPS measurements were performed using a commercial ErLEED and Phoibos 150 hemispherical energy Analyzer, and a monochromatized Al Kα X-ray source (XR 50 M) from SPECS. The pass energy was set to 50 eV for the survey scans and 20 eV for the specific regions, yielding overall spectral resolutions of 1.1 eV and 0.6 eV, respectively. The initial calibration of the energy scale and resolution of the XPS system was carried out using a Au foil (Au 4f_7/2_ at 84.0 eV (ref. 30)). The sample charging was corrected by considering the main component of metallic Ni 2p_3/2_ at 852.6 eV.^30,31^ The peak fit was performed using CasaXPS software, version 2.3.18PR1.0, by considering a symmetrical Gaussian–Lorentzian (G–L) sum with tail modifiers for asymmetric lineshape components when indicated, fixing the same full width at half maximum (FWHM) and G–L ratio for each component, and subtracting a Shirley-type background. The fit model is described in detail in the following sections. IPSA^32,33^ was carried out using the QUASES-Analyze software,^34^ taking the Ce 4d and O 1s extended regions and a bulk CeO_2_ reference sample. The inelastic electron mean free path (IMFP) through the cerium oxide matrix was calculated using the Tanuma, Powell, and Penn formula IMFP-TPP2M.^35^

## 3 Results and discussion

### 3.1 Growth mode and interface formation

Fig. 1(b) shows the XPS spectra of the Ce 3d region of the M region after the three deposition times. Black dotted lines represent the experimental data, and the fitted curves are shown in red solid lines. The complex Ce 3d spectrum of cerium oxide is associated with the strong mixing of 4f^0^, 4f^1^, and 4f^2^ configurations in the ground and final states due to the strong electron–electron correlations,^36–38^ giving rise to two “triplets” in the CeO_2_ spectra, and two “doublets” for Ce_2_O_3_. Therefore, it consists of six peaks associated with Ce^4+^ (v, v″, v‴, u, u″, and u‴, represented in blue) and four components related to Ce^3+^ (v_0_, v′, u_0_, and u′, represented in green).^39–42^ Additionally, two plasmon features (*P*_1_ and *P*_2_) are included in the Ce^3+^ spectrum, as reported for other crystalline growths.^41^ Furthermore, we have used a tail modifier to model the asymmetry of v, v″, u, and u″ components,^43^ similar to Skála *et al.*^42^ and in line with the asymmetries calculated by Kotani and coworkers.^36–38^ As an example, the fit model for the longer deposition time is shown at the bottom of the figure. For the different deposition times, all spectra show a noticeable difference in the ceria oxidation state, with a lower amount of Ce^3+^ observed as the deposition time increases. This trend can be easily followed by the presence of the peaks related to the v′ (886.0 eV) and u′ (904.5 eV) components.

Fig. 1(c) shows the XPS measurements of the Ni 2p region at the M position for the three depositions. Here, all the line shapes are similar, with the only noticeable change being a decrease in intensity as ceria deposition time increases. The spectra are labeled according to the total deposition time. The fit model for the growth after 20 min is depicted at the bottom. For the Ni 2p_3/2_, the metallic nickel consists of the main peak (Ni) and two satellites (Ni_sat_), shown in purple.^44^ Nickel oxide presents a complex electronic structure. For the NiO 2p_3/2_, it consists of a main line at ∼854.5 eV, followed by a shoulder at ∼856 eV and a satellite at ∼862 eV. Although the origin of the main line and charge-transfer satellite is well established, the assignment of the shoulder remains under discussion. Here we follow the fit model of Soriano and coworkers, who, supported by cluster model calculations, have shown how both surface and nonlocal effects contribute to the shoulder in the experimental data.^31,45^ XPS measurements at different take-off angles and different electron energies, together with calculated core-level spectra, have proven the presence of this surface component related to the screening effects of an oxygen electron at the surface.^31^ This differentiation between surface and bulk components allows for the quantification of the surface-to-bulk ratio, and more importantly, may help to understand the local environment of Ni^2+^ states. Therefore, our Ni 2p_3/2_ fit model, in orange, includes four components: a main line (NiO) and a charge-transfer satellite (NiO_sat_), and two components contributing to the shoulder, a nonlocal component (NiO_L_) and a surface component (NiO_S_).

**Fig. 2:**
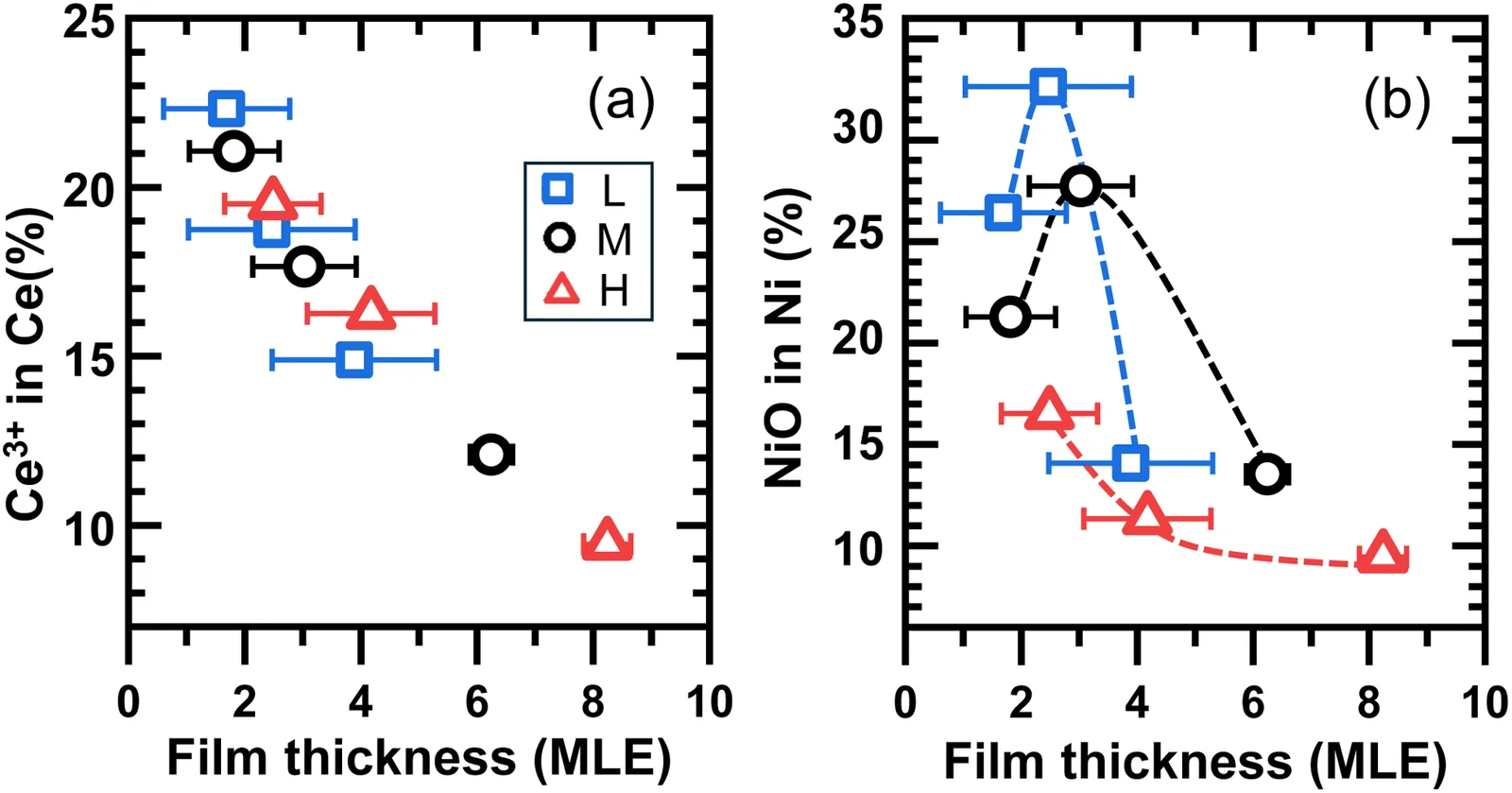
(a) Ratio of Ce^3+^ and (b) NiO for the H, M, and L positions and the three depositions as a function of the ceria film thickness, calculated using the models in Fig. 1. Dashed lines are a guide for the eye. Error bars are calculated using the standard deviation of the results obtained when calculating the film thickness for all peaks.

The amount of reduced ceria and oxidized nickel was quantified at a total of nine points, corresponding to the three different positions measured for each of the three deposition times. The results are represented as a function of the film thickness, expressed in monolayer equivalents (MLE). This thickness was estimated using Lambert–Beer's law by calculating the change in peak intensity of the film or substrate. Fig. 2(a) shows the amount of Ce^3+^ calculated considering the intensity ratio of the Ce^3+^ peaks in relation to the total ceria signal. Here, we can observe that the ceria oxidizes as its effective thickness increases, with all the measured positions following the same trend. Importantly, the Ce^3+^/Ce^4+^ ratio is independent of the growth rate and, therefore, of the relative dosage of O_2_ with respect to the amount of deposited Ce within our parameter range. This can be easily demonstrated by comparing the two points around 2.5 MLE: the H point, which corresponds to the high ceria concentration of the first growth, is only slightly more reduced than the L point of the subsequent deposition, whose total oxygen exposure is doubled compared to that of the first growth. This result indicates that the main contribution to the ceria oxidation state is the interaction with the Ni substrate.

In the case of nickel, the general trend is the opposite, with a more reduced state as the amount of ceria increases, indicating oxygen migration to the ceria film. Fig. 2(b) shows the amount of NiO, estimated by considering the total intensity of Ni and NiO components for the three positions and deposition times, plotted as a function of ceria MLE. As for the Ce^3+^/Ce^4+^ ratio, the different positions behave differently and seem to shift their NiO (%) maxima with respect to each other. For the L and M positions, the amount of NiO increases more than 5% at the second growth stage and decreases at the final step. In contrast, the NiO component decreases rapidly and continuously for the H position. Furthermore, a comparison of the L point from the second growth and the H point from the first growth (around 2.5 MLE) shows that longer oxygen exposure results in three times more NiO. Thus, NiO follows a more complex trend and appears to be influenced by oxygen exposure, extending beyond the effective thickness of ceria.

**Fig. 3:**
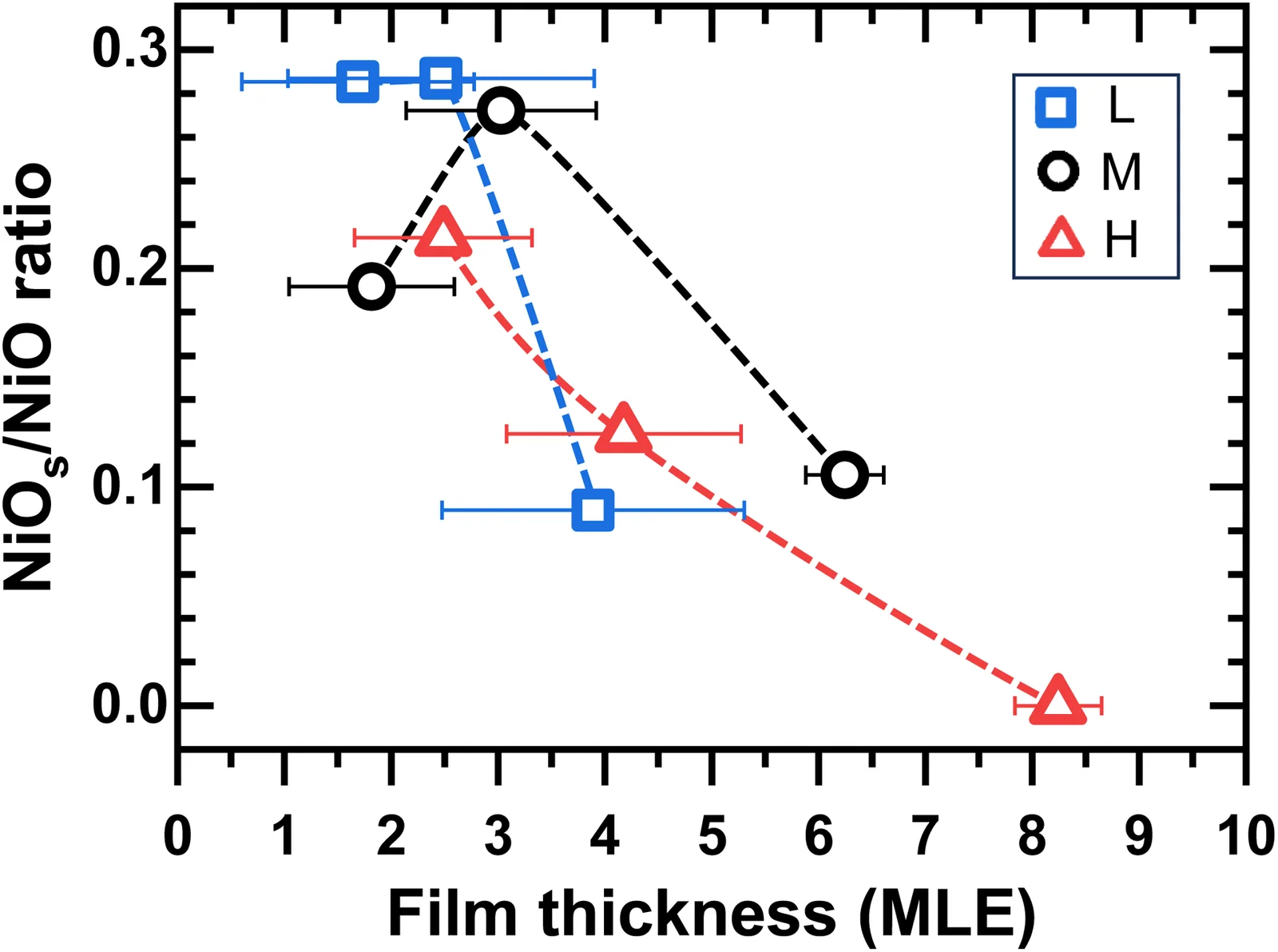
Intensity ratio between the surface component of NiO and the main component, for the three positions and the three deposition times.

Fig. 3 shows the intensity ratio between the NiO_S_ surface component and the main NiO peak, revealing different trends for each measured position. More specifically, at the L position, the surface component remains almost constant during the first two depositions before decreasing rapidly at the final stage. In contrast, the M position shows an increase in the surface component, indicating a greater proportion of the surface-to-bulk ratio. Similar to the L position, this component also decreases sharply in the final deposition stage. For the H position, the trend mirrors that of the total NiO amount, showing a continuous decrease across all deposition stages. To understand these differences, we need to consider that this surface component depends on both the oxidation of the nickel substrate and the amount of free, oxidized surface. As in the case for the overall NiO percentage, the trends for the NiO surface component contribution are not correctly explained by the MLE quantification of the ceria deposit, which assumes a homogeneous film. Instead, the presence of NiO appears to be strongly correlated with ceria growth morphology, *i.e.*, coverage and real thickness of islands, and the interaction with the Ni.

**Fig. 4:**
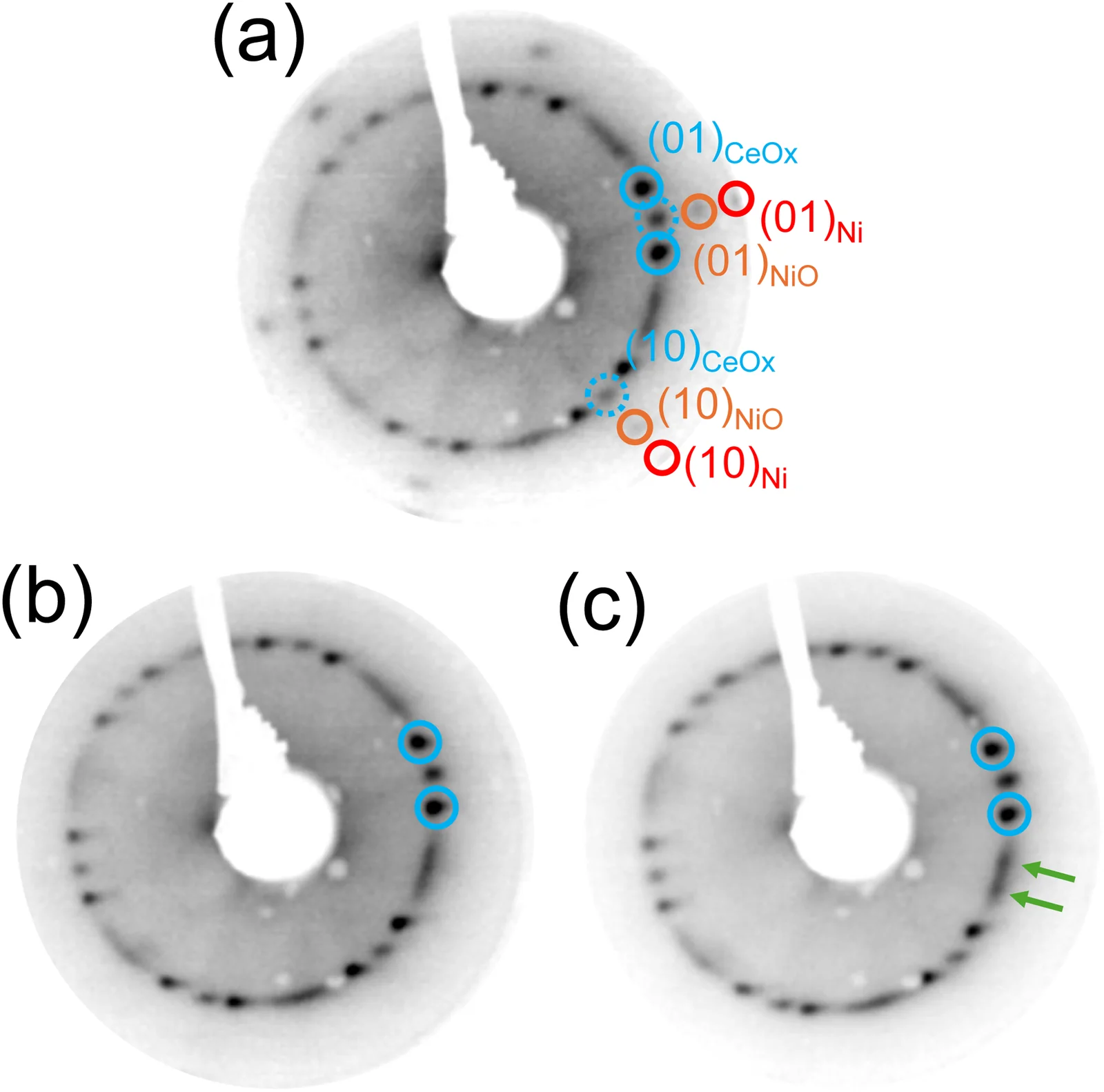
LEED images measured at 87.5 eV of the CeO_2_ deposited on Ni(111) during a total time of (a) 5 min, (b) 10 minutes, and (c) 20 minutes. The peaks associated with the CeO_*x*_(111), NiO(111), and Ni(111) are marked in blue, orange, and red, respectively. For CeO_*x*_, the dashed circle indicates 0° rotation, and solid lines mark ±11° rotations. Green arrows indicate two extra rotations that can be distinguished in the ring.

In addition to XPS data, LEED images were acquired after each deposition step at the M position to gain insight into the structural evolution of the film (see Fig. 4). Under these deposition conditions, ceria grows with a (111) orientation, forming an almost closed, faint circle with two preferential directions azimuthally rotated by 11 ± 2° (marked in solid blue in Fig. 4) with respect to the substrate spots. After the first deposition step, the spots associated with the substrate are still weakly visible, indicating that some nickel areas remain uncovered. Ni is easily oxidized under these growth conditions,^28^ forming nanostructures separated by areas of clean Ni(111). Accordingly, the noticeable difference in the lattice parameters of Ni(111) and NiO(111) allows us to distinguish two peaks in the LEED pattern, marked in red and orange, respectively, in Fig. 4(a). Using the Ni(111) lattice parameter as a reference (*a*_Ni(111)_ = 3.52 Å),^46^ the calculated lattice parameter for NiO(111) is *a*_NiO_ = 4.16 ± 0.10 Å, which is in very good agreement with previous studies on the formation of NiO films at similar conditions (*a*_NiO_ = 4.14 Å)^28^ and bulk NiO(111) values (*a*_NiO_ = 4.177 Å).^47^ For the ceria, the calculated lattice parameter is *a*_CeO_*x*__ = 5.31 ± 0.10 Å. This value is 1.8% smaller than the relaxed, completely oxidized CeO_2_ (*a*_CeO_2__ = 5.41 Å).^48^

Further cerium evaporation results in additional surface coverage, leading to the disappearance of the Ni and NiO reflections. The ceria growth follows the same pattern as before: all the features associated with the ceria intensify when increasing the deposition time, and a higher intensity is maintained for the preferential directions at ±11°. A third set of peaks becomes more intense, which is aligned along the main substrate directions. A similar phenomenon has previously been reported for ceria growth on Pt(111) at comparable temperatures,^24^ where a combination of low-energy electron microscopy (LEEM) and micro-illumination LEED showed that the (111)-oriented ceria islands grow in three rotational domains, one aligned with the substrate (0°) and two mirror domains at rotation angles of ±5°.

At the final step, the continuous circle that forms in the LEED pattern due to the CeO_2_ in between the Ni(111) spots shows the formation of more rotational domains at ∼±22°. Green arrows in Fig. 4(c) indicate the position of these new spots. Previous studies of ceria film deposition on Ni(111) also show different rotational domains.^18^ In this work, LEED patterns have demonstrated that the CeO_2_ grows in the (111) orientation, with spots occurring every 20° when deposited at lower temperatures.

**Fig. 5:**
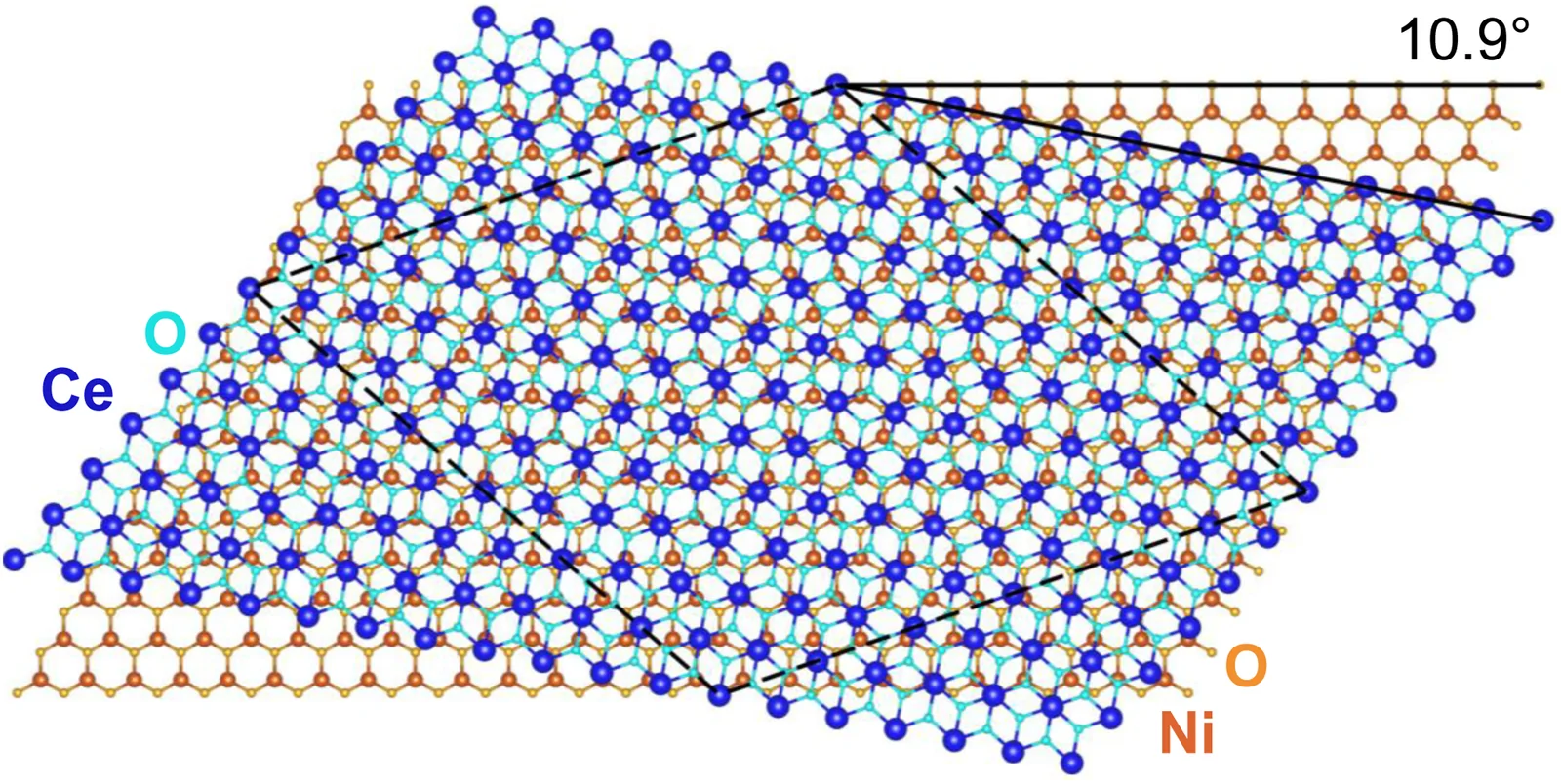
Structural model of a CeO_2_(111) trilayer on top of NiO(111), represented in blue and orange, respectively.

To understand the main rotations (at ±11°) of the cerium oxide islands, we consider the atomic structure. Fig. 5 shows an atomic O–Ce–O trilayer of CeO_2_(111) on top of NiO(111), depicted in blue and orange, respectively. Considering the lattice parameters obtained from the LEED images, the smallest coincident superstructure is found when an angle of 10.9° is formed between the two layers. This simple model can explain the preferential formation of the main spots at ±11° in the LEED patterns. However, a detailed atomic model of the CeO_2_(111)/NiO(111) superstructure requires high-resolution µ-LEED, or atomically resolved scanning probe microscopy measurements. It is worth noting that no coincidence superstructure is found when considering an atomic trilayer of CeO_2_(111) on metallic Ni(111), in accordance with previous XPS measurements and with the IPSA analysis presented in the next section.

### 3.2 Interplay of cerium oxide morphology and structure

To obtain more information about the general morphology of the ceria film and the NiO nanostructures, we studied the inelastic background of the XPS measurements. The shape of the inelastic background depends on the energy loss of the electrons from a given element and is therefore correlated with the material's distribution within the sample.^32,33^ Thus, morphological parameters such as island size, thin film thickness, and associated coverage can be obtained, thereby providing a complementary tool to the chemical characterization offered by XPS, which is particularly helpful when analyzing growth modes and evolution of buried interfaces.^34^ The reliability of the inelastic peak shape analysis (IPSA) has been successfully validated by direct comparison with microscopy techniques, such as atomic force microscopy^49–51^ and transmission electron microscopy,^52,53^ and more recently by *operando* ellipsometry.^54,55^

**Fig. 6:**
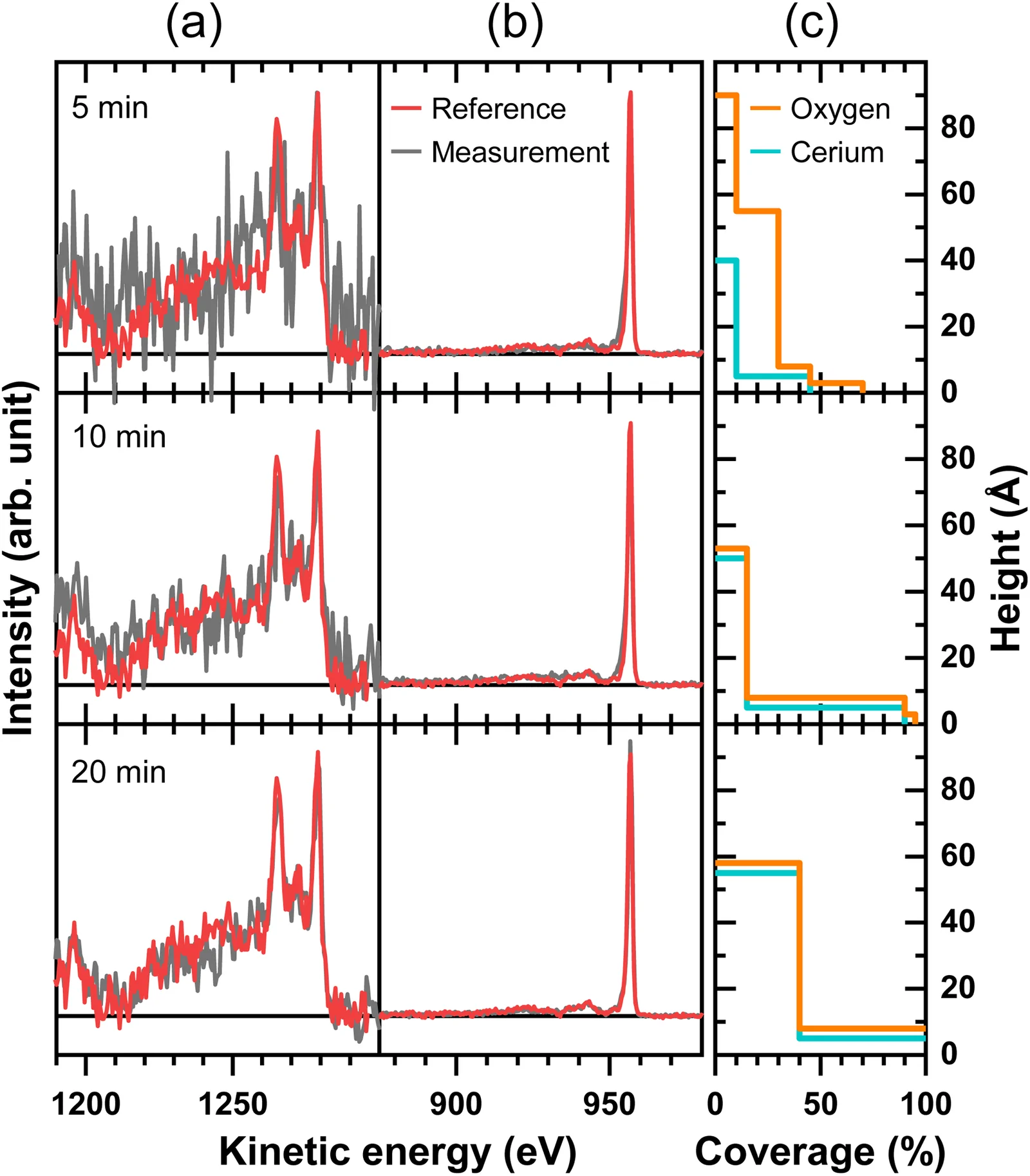
(a) Ce 4d and (b) O 1s spectrum measured at the M region for the three deposition times (in gray), and reference bulk ceria (in red) after background subtraction. (c) Island heights and coverages for cerium and oxygen signals, obtained from the regions in (a) and (b).

To gain insight into the morphology of the ceria deposit, we analyzed the Ce 4d region. We note that the Ce 3d region cannot be used, as it directly overlaps with the Ce MNN Auger peaks when measuring with Al Kα. More importantly, the inelastic background is modified by the Ni 2p region being so close in energy. Fig. 6(a) shows the Ce 4d spectra at the M position for the three deposition stages after background subtraction (gray line) compared with a reference of bulk CeO_2_ (in red). The reference sample is a 20 nm thick bulk ceria film grown by ALD on a silicon substrate. Its thickness ensures that only ceria peaks are visible in the XPS measurements, confirming its bulk morphology. As previously discussed, the nickel oxidation state undergoes significant changes during the various deposition stages. To understand the origin of these changes, the O 1s region is also studied (Fig. 6(b)). While the IPSA of the Ce 4d region provides information only on the distribution of Ce cations regardless of their oxidation state (Ce^3+^ or Ce^4+^), as the ceria structure presents almost the same density of Ce atoms, the O 1s region allows us to analyze the distribution of oxygen in both the ceria and the Ni substrate. This combined approach facilitates the creation of a complete morphological model of the growth mode for both CeO_*x*_ and NiO. It is important to note that since the O 1s reference is also taken from the bulk CeO_2_, a slight deviation from the actual values of thickness and total coverage for the NiO is expected. However, this does not affect the general trends and the determined morphology.

Fig. 6(c) shows the resulting height and substrate coverage for cerium and oxygen. We observe that two types of ceria islands (blue lines) form for the three deposition times, with their total coverages increasing with the deposition time. At the first deposition stage, the ceria covers only half of the substrate's surface, as expected from the LEED image in Fig. 4(a), where the reflections associated with the Ni and NiO are visible. Tall islands with a height of 40 Å cover around 10% of the substrate, while around 35% is covered by islands that are only about 5 Å tall (*i.e.*, compatible with a mixture between one and two O–Ce–O trilayers in the [111] direction). As the deposition time increases, the ceria coverage reaches approximately 90% of the substrate. During this stage, the tall islands increase in height, reaching 50 Å, but their coverage increases only slightly. In contrast, the small islands present the opposite behavior: they now cover 75% of the substrate without increasing in height. At a total deposition time of 20 min, the substrate is completely covered. The tall islands increase slightly in height (55 Å) but show a significant increase in coverage, occupying 40% of the substrate. The rest of the surface is covered by the small islands, which have not increased in height.

The distribution of oxygen, and consequently the morphology of the oxygen-rich structures, is more complex. Similar to the ceria, the oxygen also forms a step-like structure, but with greater heights and coverage. This result indicates that the oxygen is not only part of the ceria islands but is also incorporated into the Ni substrate. At the first deposition stage, the oxygen structure shows a height of 90 Å for 10% of the coverage, which coincides with the coverage of the tall ceria islands. Since the oxygen is also part of the ceria islands, and we know that these islands are partially defective in oxygen, this result indicates that a portion of the Ni substrate is oxidized below them. Up to a coverage of 45%, where the small ceria islands end, the oxygen shows heights of 55 Å and 8 Å, indicating varying depths of NiO beneath these islands. Finally, 25% of the free nickel surface is oxidized, leaving 30% of the surface oxygen-free. Therefore, this analysis explains the presence of Ni and NiO spots in the LEED pattern in Fig. 4(a).

At a total deposition time of 10 minutes, in the second stage, the oxygen distribution mainly follows the cerium, indicating the formation of NiO predominantly below the ceria. The oxygen structure is now about 3 Å higher (*i.e.*, compatible with the Ni–O layer thickness of about 2.4 Å in the [111] direction) than the ceria for both types of islands, indicating that the oxygen is mainly forming a layer below the ceria. In addition, an oxygen layer of 3 Å is covering 5% of the free Ni surface, leaving the other 5% of metallic Ni at the surface. For the last growth stage, the oxygen distribution completely follows the ceria structures, indicating a homogeneous layer beneath the ceria film, together forming a CeO_2_(111)/NiO(111)/Ni(111) heterostructure.

**Fig. 7:**
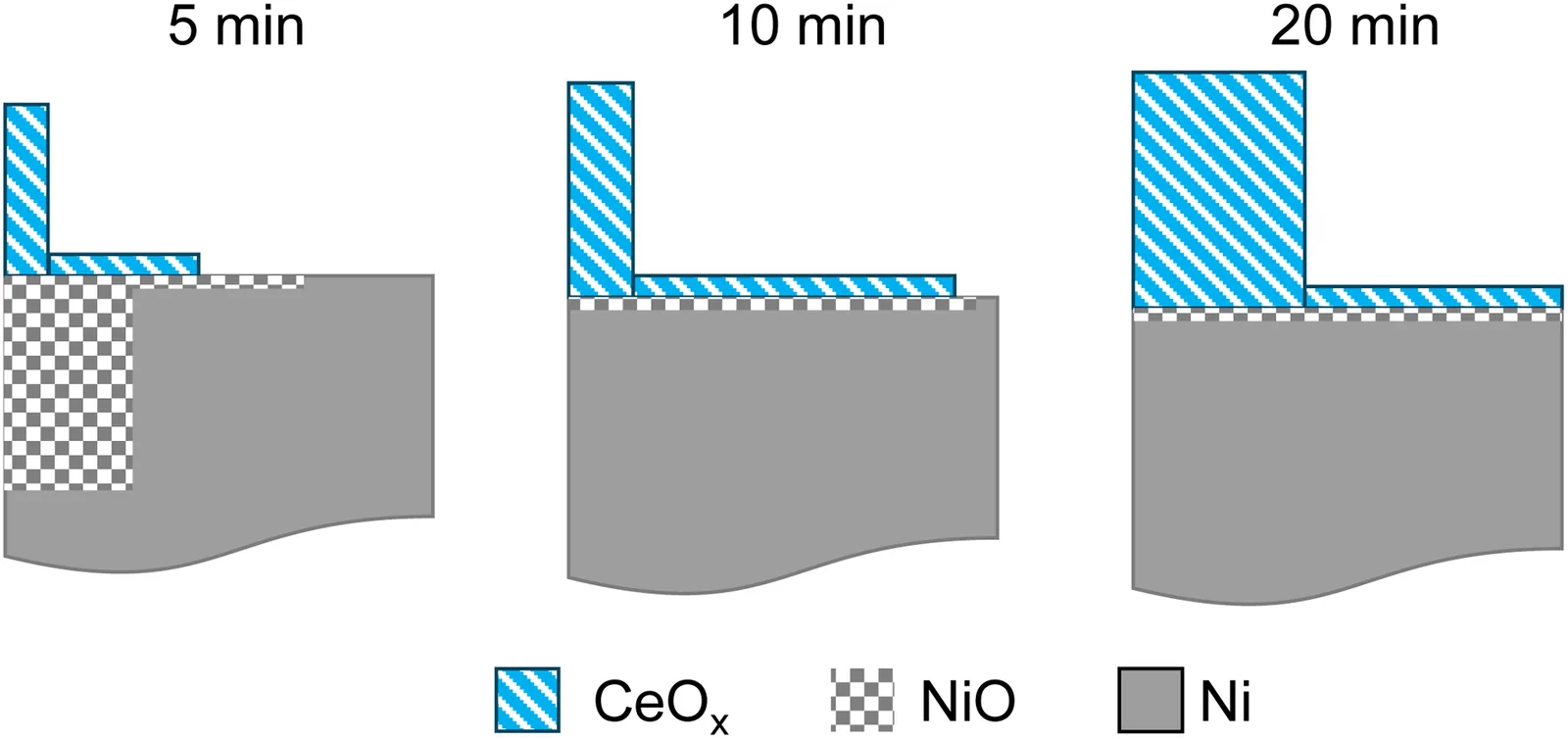
Schematic representation of the IPSA results for the M position at the three deposition stages, showing the two types of ceria islands and their relation to the underlying nickel oxide.

The results are better rationalized in the schemes in Fig. 7, where the two types of ceria islands (in blue and white) are depicted on top of the substrate, with the heights and the coverage reflecting the results of Fig. 6(c). White squares in the substrate indicate the nickel oxide, which primarily forms a thin layer between the ceria islands and the metallic substrate. This model shows that, upon further cerium deposition, oxygen migrates from the oxidized substrate to the ceria islands, which aligns with the XPS results discussed earlier. Furthermore, it can also explain the complex behavior of both the percentage of NiO and the NiO_S_ component in terms of ceria total coverage and height. Initially, the Ni(111) surface is heavily oxidized, a process apparently catalyzed by the deposited cerium metal; however, the extended growth of CeO_*x*_ results in substrate reduction, with the nickel oxide underneath acting as an internal source of oxygen for the ceria deposit. Only an ultrathin layer is stabilized at the interface in parallel to Ce^3+^ states, as discussed before, with the amount of reduced ceria as a function of the deposited MLE. This effect is similar to the Ni^2+^ promotion of metallic Ni nanoparticles deposited on the ceria surface.^4,8,12^ Moreover, this CeO_*x*_/NiO/Ni interface is confirmed by the gradual decrease of the NiO_S_ component. The growth of ceria on top of NiO acts similarly to a new layer of the metal oxide. Although the fine structure of the Ni 2p for the NiO layer will likely be slightly modified by the presence of ceria and metallic Ni at the top and bottom, respectively, this component can still be used in the fit to track the evolution of the interfacial region. In particular, further experiments exploring the catalytic behavior of ceria/Ni catalysts can help distinguish the likely different roles of surface Ni^2+^ states and those stabilized at the interface.

**Fig. 8:**
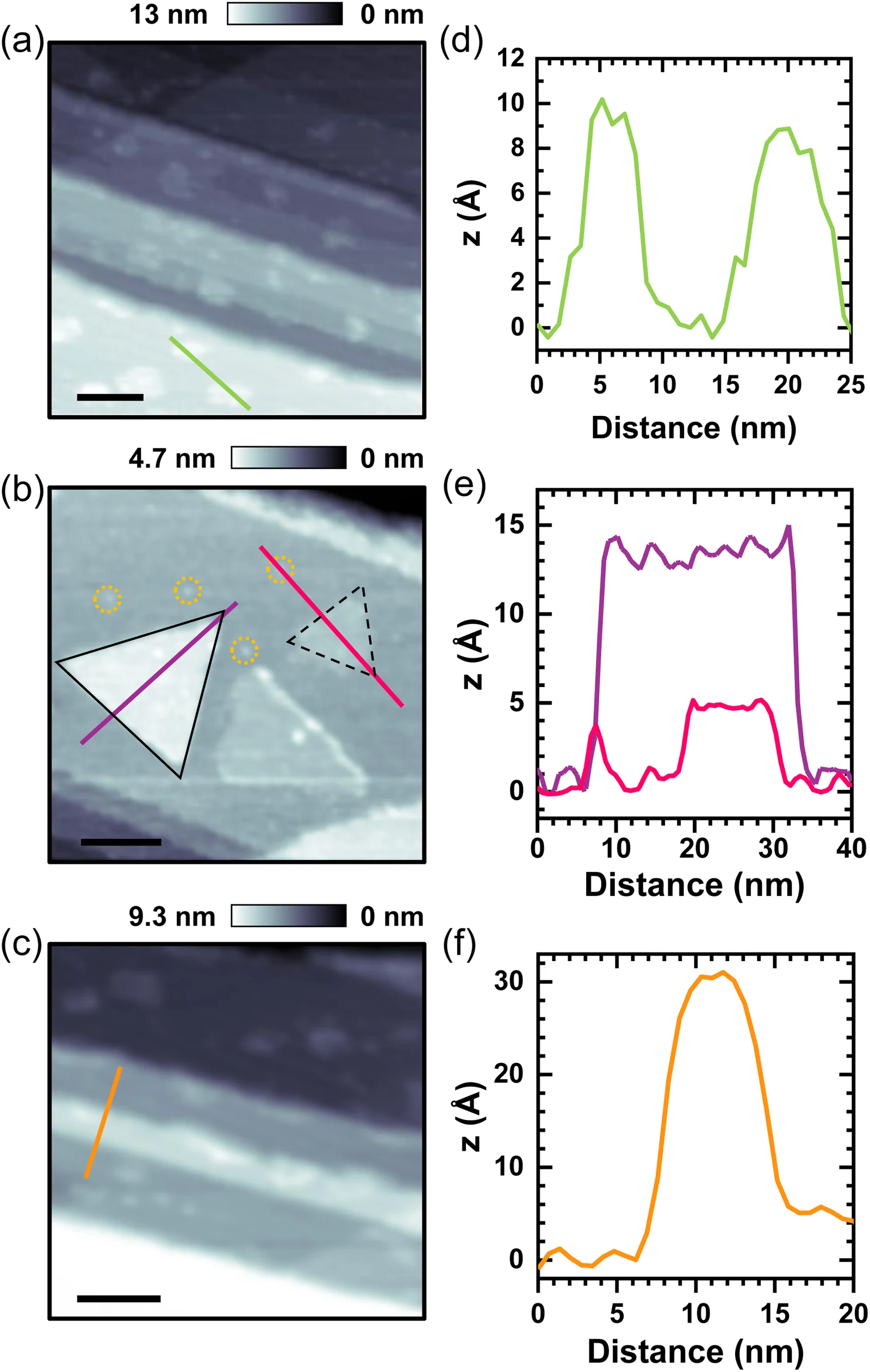
(a–c) STM images measured at 2 V and 60 pA. Horizontal scale bars are 15 nm long. Yellow circles in (b) indicate small islands, and solid and dashed triangles highlight islands with a relative azimuthal rotation of ∼22°. (d–f) Height profiles corresponding to the color-coded lines in (a–c).

STM images acquired for the lowest growth time in the M position confirm the coexistence of two distinct island morphologies. The surface is predominantly covered by thin, small islands less than 10 nm in size, growing on the terraces, like the ones shown in Fig. 8(a). Most of these small islands do not exhibit well-defined shapes. This high density of irregularly shaped islands can lead to random azimuthal orientations, which manifests in the LEED patterns as a high number of rotational domains or a continuous ring, as well documented for cerium oxide growth on other substrates.^16,17,21,56^ While most islands lack a well-defined morphology, a small fraction exhibit the characteristic triangular shape of CeO_2_(111) (Fig. 8(b)). On typical hexagonal substrates, epitaxially grown CeO_2_(111) yields two rotational domains with a difference of 180°.^17^ In our case, in addition to these mirror rotations, pairs of triangular islands are also readily found, with a difference in azimuthal rotation angle of ∼22° (indicated by the solid and dashed lines in Fig. 8(b)). Thus, the presence of these orientations matches the observation that the main spots in the LEED patterns are found at ±11°, implying that a substantial fraction of the irregular islands likely share these two orientational domains. Additionally, small islands of around 1 nm in diameter (marked by yellow circles in Fig. 8(b)) indicate the nucleation of new ceria islands in the first stages of growth. Together, these observations closely parallel the growth characteristics of ceria on Pt(111), where the islands grown on the Pt(111) terraces exhibit one of the two rotated orientations of ±5°, while the ones nucleated at the step edges appear aligned with the main substrate directions.^24^ Analogously, for the present Ni(111) system, we tentatively correlate the ceria structures decorating the substrate step edges (Fig. 8(d)) with the unrotated (0°) diffraction spots identified in the LEED images.

Height profiles of the STM images confirm the presence of two types of islands. The thin islands present at the substrate steps show a height between 1 and 4 O–Ce–O layers (Fig. 8(d and e)). These islands correspond to the thin islands in the IPSA that cover most of the surface for the lower growth time. At the same time, taller ceria structures, tens of Å in height (Fig. 8(f)), are found at the step edges, primarily decorating and growing along the steps, and are comparable in height to the tall islands identified in the IPSA.

**Fig. 9:**
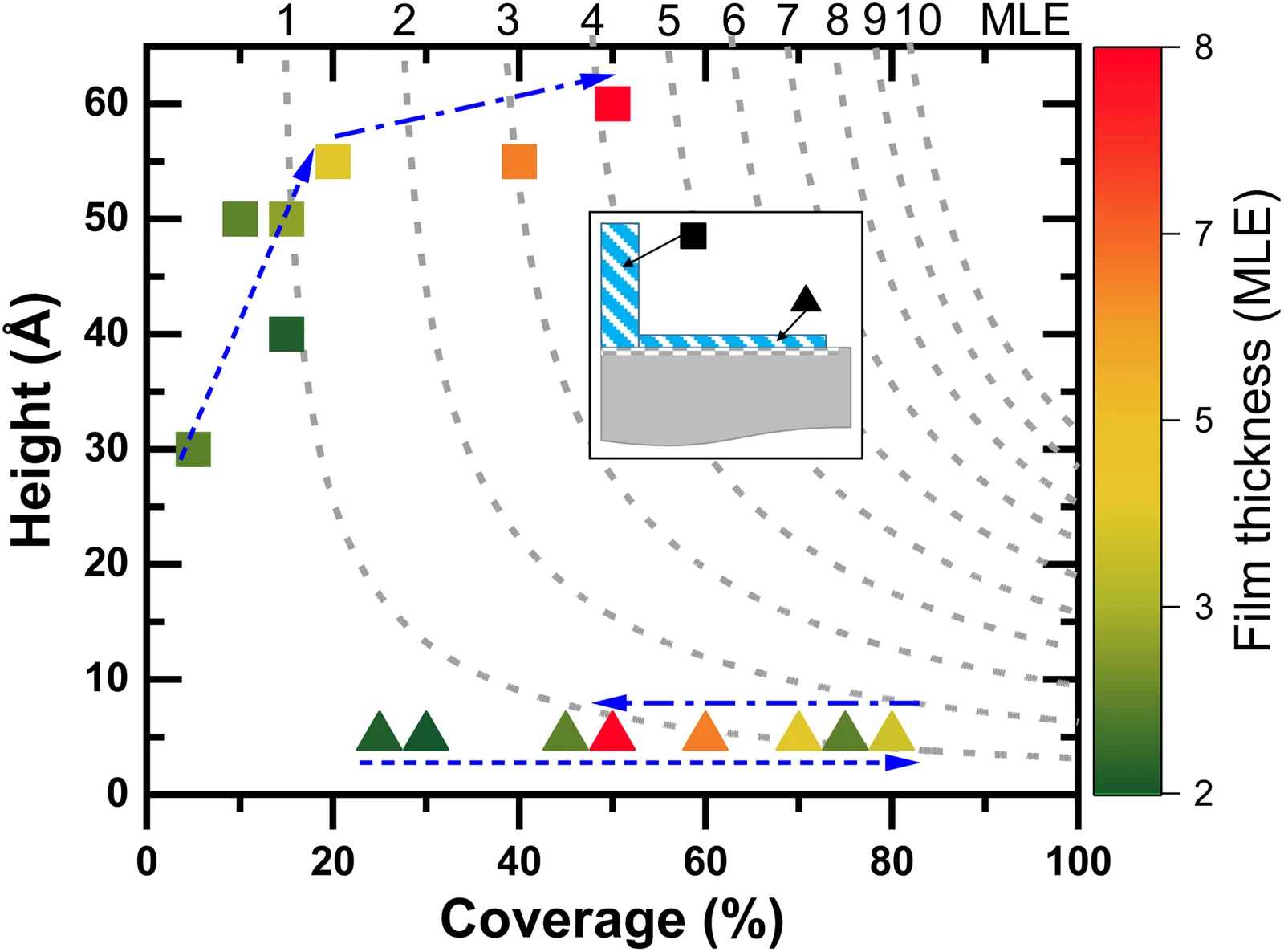
Results of the inelastic XPS peak shape analysis, showing the height of the CeO_*x*_ as a function of the coverage of the substrate. Triangles (squares) refer to flat (tall) islands. The color scale indicates the calculated layer thickness of the CeO_*x*_ film assuming a layer-by-layer growth. Blue arrows indicate the two growth regimes. Dashed gray lines correspond to constant MLE curves.

A more complete picture emerges when the IPSA is applied to the inelastic XPS peaks for all sample positions and the three growth stages. Fig. 9 shows the height of the tall islands (squares) and small islands (triangles) as a function of the substrate coverage. The colors of the symbols correspond to the total film thickness estimated assuming a layer-by-layer growth (see color bar on the right axis). Two regimes can be distinguished. First, the smaller islands, which form a wetting layer, rapidly increase in coverage, eventually reaching 80% of the surface (blue dashed line). Meanwhile, the thick islands are increasing rapidly in height (from 30 Å to 50 Å) and at a noticeably slower rate in coverage, remaining below 20% coverage.

At this point, we propose that the nucleation and growth of the two different types of ceria deposits follow a partial Stranski–Krastanov-like growth mode, closely related to the initial state of the oxidized Ni(111) surface. Previous reports at similar temperatures have indicated that Ni(111) initial oxidation is stronger at the steps, forming a NiO(111)-like film on the terraces consisting of nanosized domains.^28^ Therefore, in our scenario the CeO_*x*_ would nucleate at the substrate steps, extending first as a thin layer along the step edges before rapidly transitioning to a 3D growth mode, increasing mainly in height, as illustrated in Fig. 9. Simultaneously, a ceria wetting layer would nucleate on the terraces, forming (111)-oriented, small, thin islands that grow laterally in size over the NiO(111) layer. The majority of the ordered ceria domains adheres to the main ±11° azimuthal alignment relative to the main directions of the underlying Ni(111) lattice. Once the surface is completely covered by the ceria wetting layer, the growth mode deviates from the initial trend in Fig. 9 (blue dashed lines). At this stage, the accumulation of elastic, compressive strain energy within the wetting layer (reflected in the measured ceria lattice parameter in LEED) drives a transition towards 3D growth, exhibiting Stranski–Krastanov-like behavior. As deduced from the IPSA (Fig. 9), these 3D structures increase rapidly in coverage while their height increases at a noticeably slower rate. Consequently, the wetting layer coverage decreases (dash-dotted blue lines). At 8 MLE, which is the highest deposit measured, both types of islands cover 50% of the substrate, and the tall islands reach 60 Å in height. Hence, this scenario would also readily explain the similar coverage of the ceria and NiO islands in the later growth stages of Fig. 6(c).

## 4 Conclusions

In this study, we provide a detailed analysis of the growth mode of the CeO_*x*_/Ni(111) system at moderate temperatures. Our findings reveal the complex behavior of this system at the interface, which defines the morphology and the oxidation state of the ceria film. Using XPS, we demonstrate a clear correlation between the oxidation state of the ceria and its effective thickness. As the film grows, the proportion of reduced Ce^3+^ species decreases, a trend independent of oxygen exposure during growth. This finding highlights the significance of the oxide–support interaction, which is the primary driver for the ceria's oxidation state.

The detailed analysis of the NiO components, combined with the use of the IPSA method to analyze the inelastic background of the XPS spectra, provided critical insights into the underlying structural evolution during cerium oxide growth. Combined with LEED analysis, this morphological study confirms the formation of a CeO_*x*_(111)/NiO(111)/Ni(111) interface. By combining STM measurements and IPSA, we propose a partial Stranski–Krastanov-like growth mode, where an initial ceria wetting layer is formed on the terraces while taller cerium oxide islands nucleate and grow at the step edges. The LEED patterns and STM measurements show that the cerium oxide wetting layer is preferentially stabilized with an azimuthal lattice rotation of ±11° relative to the underlying ultrathin NiO(111) layer. The distinct azimuthal rotation of the cerium oxide lattice results from coincidence lattice formation with the NiO(111) interface rather than with the metallic Ni(111) substrate. Upon further deposition, the residual strain in the wetting layer is likely the reason for the subsequent transition from 2D to 3D growth.

Initially, the Ni metal substrate is oxidized to form a comparatively thick NiO(111) layer and then acts as an internal supply of oxygen in subsequent cerium oxide growth. This NiO(111) interlayer then exerts a templating effect for subsequent ceria growth, with pronounced structural consequences for the CeO_*x*_(111) structure and interfacial properties. These findings suggest that this system can serve as a robust model for understanding catalytic processes where dynamic oxygen mobility and structural-chemical surface–interface interactions are expected to modulate the chemical activity.

## Author contributions

Conceptualization, R. Sánchez-Barquilla, C. Morales, J. I. Flege; methodology, R. Sánchez-Barquilla, D. Guttmann, C. Morales; validation, D. Guttmann; formal analysis, D. Guttmann; investigation, R. Sánchez-Barquilla, D. Guttmann; resources, J. I. Flege; data curation, R. Sánchez-Barquilla, D. Guttmann, C. Morales; writing—original draft preparation, R. Sánchez-Barquilla; writing—review and editing, C. Morales, J. I. Flege; visualization, R. Sánchez-Barquilla, D. Guttmann; project administration, J. I. Flege; funding acquisition, J. I. Flege. All authors have read and agreed to the published version of the manuscript.

## Conflicts of interest

There are no conflicts to declare.

## Data Availability

Primary data of this article are available at the repository of the APH group at BTU upon reasonable request.
